# Self-organization at the first stage of honeycomb construction: Analysis of an attachment-excavation model

**DOI:** 10.1371/journal.pone.0205353

**Published:** 2018-10-24

**Authors:** Takayuki Narumi, Kenta Uemichi, Hisao Honda, Koichi Osaki

**Affiliations:** 1 Graduate School of Sciences and Technology for Innovation, Yamaguchi University, Ube, Yamaguchi, Japan; 2 Department of Mathematical Sciences, Kwansei Gakuin University, Sanda, Hyogo, Japan; 3 Graduate School of Medicine, Kobe University, Kobe, Hyogo, Japan; 4 Center for Biosystems Dynamics Research, RIKEN, Kobe, Hyogo, Japan; Arizona State University, UNITED STATES

## Abstract

Honeybees construct nests that consist of regularly arrayed hexagonal cylinders. In the first stage of honeycomb construction, they build a linear sequence of tetrapod structures that form the basis of the comb. However, considering their physiological limitations, it is unknown how honeybees produce that initial pattern. Herein, in an attempt to understand the mechanisms of honeycomb construction, we propose an agent-based model, the attachment-excavation model, in which worker honeybees are classified into attachers who secrete and attach wax, and excavators who excise the attached wax. The model assumes that workers instinctively refrain from digging through the thin parts of a wax cluster. We then conduct two-dimensional (2D) simulations that show how a tripod pattern can be seen as a projection of tetrapods onto a plane. The simulation results show that the tripod pattern emerges due to competition between the attachers and excavators. As time advances, the isotropic wax growth causes the tripods to connect planarly. Because the homogeneously broadened structures do not match that of a natural comb, we employ anisotropic wax growth to obtain a linear sequence of constructed tripods, thus suggesting that anisotropy is a significant contributor to the first stage of honeycomb construction. From our simulation results, we conclude that honeybees utilize self-organization to achieve complexity during the first stage of honeycomb construction. It is anticipated that the results of our study will provide insights into how complexity can be achieved within a hierarchy.

## Introduction

Patterns exist in all areas of nature, from microscopic to macroscopic levels. Complex patterns can appear without the use of top-down methods. One bottom-up approach is the self-organization formation process of higher-level order that spontaneously arises out of local interactions among lower-level components. Self-organization is observed in both physicochemical [1] and vital systems, such as cellular differentiation [2], stripes on animal skins [3], cell biology [4], and cytosystems dynamics [5]. Living organisms, in particular, show numerous self-organized pattern types, such as the growth of bacterial colonies, the synchronized light emissions of fireflies, and the swarm dynamics of fish and birds [6–8]. In addition, some construction processes involving social insects such as ants (*Temnothorax albipennis*) [9] and termites (*Macrotermes bellicosus*) [10] can be at least partially explained in terms of self-organization. In this paper, we propose a novel viewpoint by which the first stage of honeycomb construction can be understood as self-organization.

*Apis mellifera*, also known as western honeybees, are a leading example of social insects. They live communally and care cooperatively for their young. The structure of honeybee nests consists of double-sided regularly spaced cavities. The axes of these cavities appear to be almost horizontal, but they actually slope slightly upwards toward the open ends [11]. Each hole is created in the form of a precise hexagonal prism. Honeybee nest construction, including the process by which honeycombs are made from the wax secreted by worker honeybees, has long attracted scientific interest [12].

There are two primary viewpoints on how the frames become hexagonal. One is that the precise structure is simply a result of the law of physics. Pirk et al. argued that the bees’ body heat increases the temperature in the vicinity of the cylindrical holes until the wax reaches a liquid equilibrium state, after which simple mechanical surface tension causes the hole frames to be hexagonal [13]. Meanwhile, Karihaloo et al. proved that, under the liquid equilibrium hypothesis that results from surface tension, the softened wax could be thinned so that the hexagonal frame would appear spontaneously [14]. However, Bauer and Bienefeld showed that the bees constructing honeycomb do not heat the wax to a temperature that would allow it to reach a liquid equilibrium state [15]. In addition, Oeder and Schwabe stated that any wax flows which could influence the cell geometry do not occur [16]. The other viewpoint posits that honeybees are competent engineers acting under simple rules. Oldroyd and Pratt argued that eusocial bees develop cells under simple rules during cell building and showed that natural selection for small changes in these rules could generate divergent nest structures [17]. Furthermore, Nazzi carried out a numerical simulation with simple behavioral rules and then showed regularly arranged quadrangular cells under a growing cell wall [18]. Although it remains debatable as to which viewpoint is plausible, we consider that honeybees are engineers utilizing self-organization. We should then configure simple behavioral rules of honeybees to study honeycomb pattern formation.

**Fig 1:**
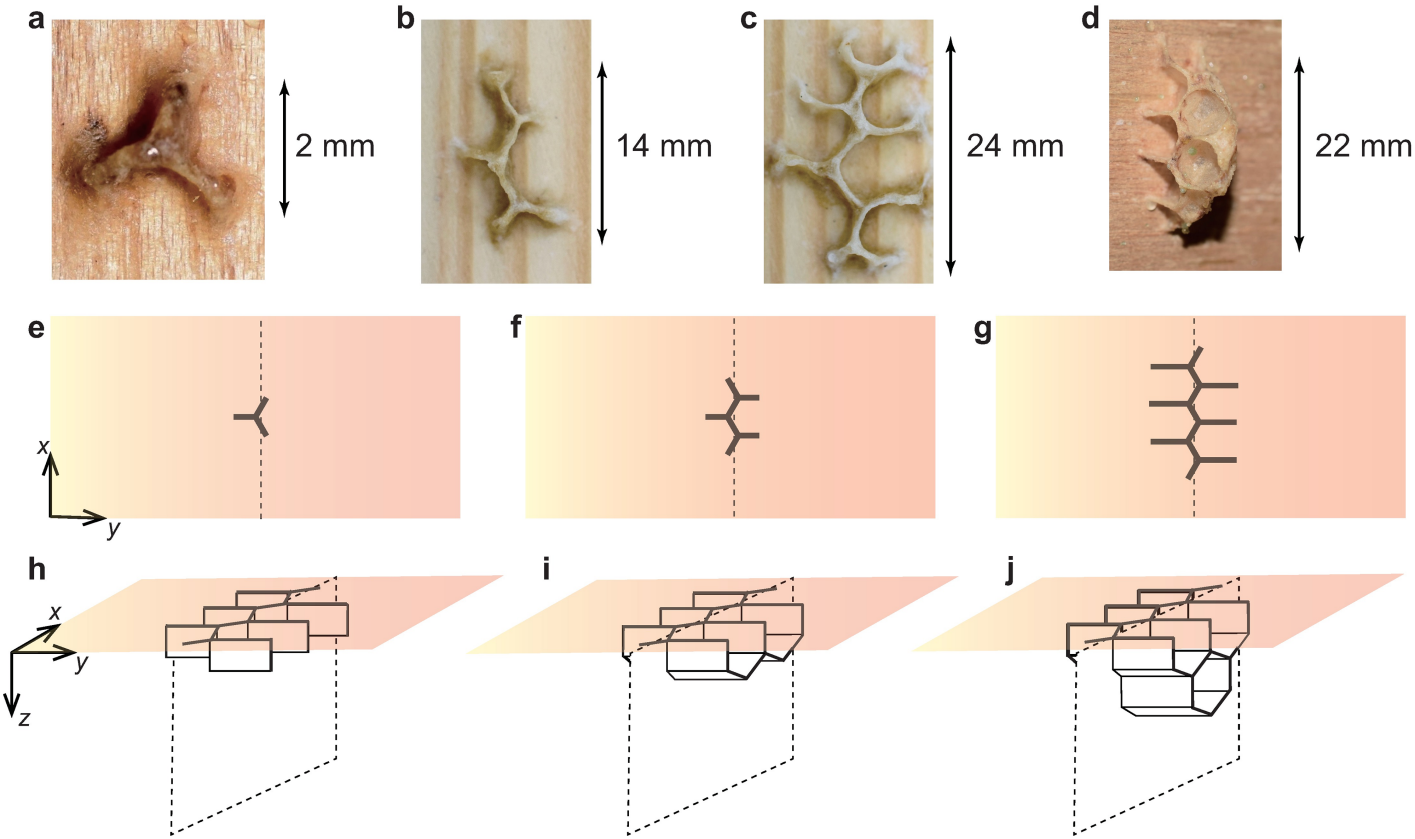
Honeycomb construction. Actual structures made of beeswax on a wood ceiling (a-d) and schematic honeycomb images (e-j) taken during the first stage of the hive construction process, where panels e-g represent two-dimensional (2D) patterns on a plane (e.g., ceiling), and panels h-j represent 3D patterns. We consider the tetrapod structure (a), the size of which is of a similar order to that of a honeybee, to be the basic building block. On the plane, several tetrapods connect horizontally (b) in one direction (*x*-direction), which then elongates (c) in the perpendicular direction (*y*-direction). The structure also grows (d) in the vertical direction (*z*-direction). The schematic images e-g correspond to a-c, respectively. Thus, the pattern grows simultaneously in each direction.

The mechanism by which honeybees construct honeycomb cells in such precise order is still an open discussion. Before construction begins, honeybees swarm onto spaces such as tree hollows, attach themselves to the ceiling in the form a hemispherical ball, and then begin constructing honeycombs within the ball. Initially, the wax is attached to the ceiling, and construction proceeds downward in the direction of gravity (Fig 1a–1c). A 3D structure is achieved as workers continue attaching wax in successive layers (Fig 1d). If honeybees are regularly aligned in the ball when constructing their nest, the precise order should appear easily. However, close observations of workers engaged in nest construction show that this is not always the case. In fact, our observations show that several workers often attach wax in turns when building a single cell (see S1 Video).

In order to understand the origin of these regular arrangements, we focus our attention on the initial structure of the honeycomb. As shown in Fig 1a and 1e, in the early phase of the construction process, the workers on the ceiling make *tetrapod* structures. These structures can be treated as the basic building block in honeycomb construction. The linear connection of several tetrapods on a plane becomes the bottom face of the honeycomb (Fig 1b and 1f), and each branch is simultaneously elongated (Fig 1c and 1g). As shown in Fig 1h, which is the stereoscopic view of Fig 1g, the structure also grows in the vertical direction. In Fig 1i and 1j, the tetrapods appear as part of the frame of honeycomb cells. Therefore, a clarification of the tetrapod formation mechanism can be expected to shed light on the first stage of the honeycomb construction process. Herein, we propose an agent-based model, which we have named the *attachment-excavation model*, to clarify the tetrapod construction from a self-organization viewpoint.

## Methods

### Overview

The attachment-excavation model is proposed as a way to understand the mechanisms of honeycomb construction; in this paper, especially the first stage of construction. At the most basic level, there are two types of workers: *attachers*, who secrete and attach wax, and *excavators*, who excise the attached wax using their mandibles. The two entities included in the model are the attached wax placed by the attacher, and excavation zones (EZs) in which the excavators remove the attached wax. In nature, it is likely that each worker is capable of performing both roles, but in our model, their functions are clearly divided to simplify role clarification.

We explain the 2D model simulations in what follows. However, it should be noted that the concept can be easily extended to 3D cases. Since the tetrapod structure is projected onto 2D space as a *tripod*, we will aim at proposing the minimum assumptions necessary for the emergence of 2D tripods.

### Time unit

We will consider a 2D system whose size is *l*_*x*_ × *l*_*y*_. As the initial model condition, a fixed amount of wax is placed at the center of the system. In nature, the wax is attached in a number of places at the beginning of honeycomb construction. In our model, we observe the growth of one such comb because our focus is on the first stage of the honeycomb construction, which is not affected by other locations.

The attachers move freely within the system, each secreting one dollop of wax per unit time. The wax is added to the boundary of randomly selected preexisting wax. In nature, since worker honeybees operate in swarms, two or more workers will inevitably supply wax simultaneously at different volumes at different points. In contrast, our model presumes that a worker attaches a fixed amount of wax to a single point on the boundary at each step. Thus, the time unit is regarded as the supply interval average.

### Attacher and wax growth

Instead of tracking the motions of the attachers, this model follows wax growth, the dynamics of which are simulated by the Eden growth rule [19]. The system domain is divided into *N*_*x*_ × *N*_*y*_ lattice cells, where each cell has size Δ*x* × Δ*y* with Δ*x* = *l*_*x*_/*N*_*x*_ and Δ*y* = *l*_*y*_/*N*_*y*_. The presence of wax in the system is expressed by a binary value assigned to each lattice cell. A lattice cell filled with wax is designated as being in the on-state, whereas an empty lattice cell is designated as being in the off-state. Therefore, the adherence of secreted wax is represented by switching from the off-state to the on-state, and the attached wax total forms a cluster of the on-state lattice cells. Once a lattice cell enters the on-state, it maintains that state until excised by the excavators. The Eden growth rule states that the off-state lattice cells around the on-state lattice cells are candidates for supplied wax at each step, after which a lattice cell selected randomly from the candidates is switched on at the next time step.

### Excavators

Since the Eden-type wax growth process does not result in a honeycomb, wax extraction is also necessary for the emergence of tripods. In our model, the excision mechanism is realized by the excavators, which move freely in the system and excavate superfluous attached wax. Here, we must emphasize that the excavators do not consciously intend to construct regularly arranged honeycomb cells; they simply remove wax based on a number of simple rules.

Based on our observations, it appears that excising plays an even more fundamental role in tetrapod construction. For example, in an earlier study, one of the authors et al. observed that a piece of colored wax that was smeared on a honeycomb under construction was removed and redistributed to other places in the honeycomb away from its original placement location [20]. This clearly suggests that honeybees not only remove excess attached wax, they transport it to other locations where it is needed to construct their nest.

**Fig 2:**
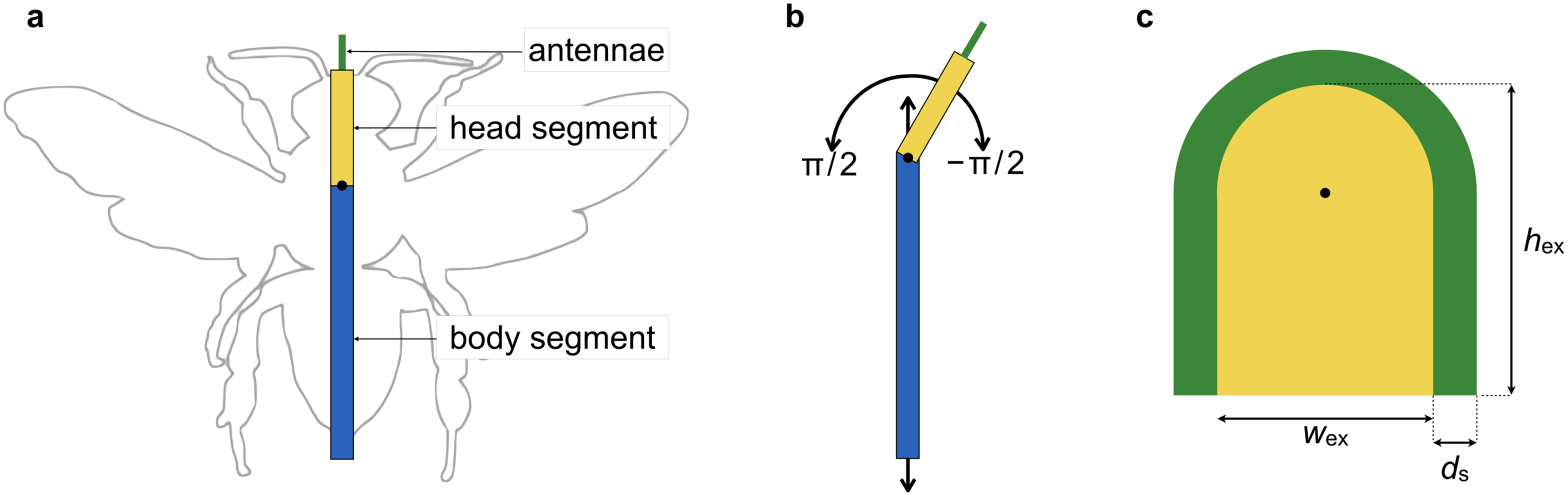
Excavator. (a) An excavator on the silhouette figure of a honeybee. The excavator consists of a body segment (blue), head segment (yellow), and the antennae (green). Hereafter, the dual antennae are treated and referred to as a single organ. The black circle indicates the connection point between segments. (b) Excavator movement. The head segment can rotate around the connection point in the range of −*π*/2 to *π*/2. The excavator can move forward and backward along the axis of the body segment. (c) Excavation zone (EZ). The yellow region indicates the EZ in which the attached wax is removed, and the green band whose width is *d*_s_ shows the area detected by the excavator’s antennae. The black circle indicates the rotation center.

The excavator worker is modeled as two connected segments (head and body) and the antennae as shown in Fig 2a and 2b. The head segment can rotate within ±*π*/2 around the connection point. Note that the head segment can rotate semispherically in 3D cases. The front edge of the head is the jaw that excises the excess attached wax. The excavator moves forward and backward along the axis of the body segment. When it turns, the body segment rotates around the connection point.

Honeybee antennae not only detect objects, they also sense temperature, humidity, and odor. Although their precise function has yet to be fully clarified, they obviously play a significant role in honeycomb construction. For example, Martin and Lindauer found that bees whose sense of touch was eliminated by the removal of antennae built imperfect honeycombs consisting of double-thickness cavities and/or wax walls with holes [11, 21].

In our model, excavator antennae have the following two abilities. One is the ability to recognize nearby locations where the wax has been attached, which allows worker honeybees to construct their nests in total darkness, and the other is the ability to measure the depth of a wax cluster from just one side. This ability prevents them from penetrating a cluster thinner than a length *d*_w_. Although it has yet to be experimentally proven whether the worker bees have measurement abilities, Martin and Lindauer have suggested that their antennae detect depth from one side by measuring the local strain of wax walls [21].

### Excavation zone

We introduce the idea of EZs in which excavators remove excess attached wax. We track the dynamics of the EZs instead of the excavators themselves. Each EZ is the area covered by the head segment when the excavator moves forward and backward while allowing the head segment to rotate within a restricted angular range. Consequently, as shown in Fig 2c, each EZ consists of a box with a semicircle in front. Every EZ has the same size and form, which remain unchanged during movement. The length *h*_ex_ is independent of the length of the body segment, and corresponds to the depth into which the excavator digs. When an EZ turns, it rotates around the center of the semicircle, corresponding to how the excavators rotate. Since the excavator immediately removes the excess wax, it is not reattached within that EZ.

### EZ dynamics

**Fig 3:**
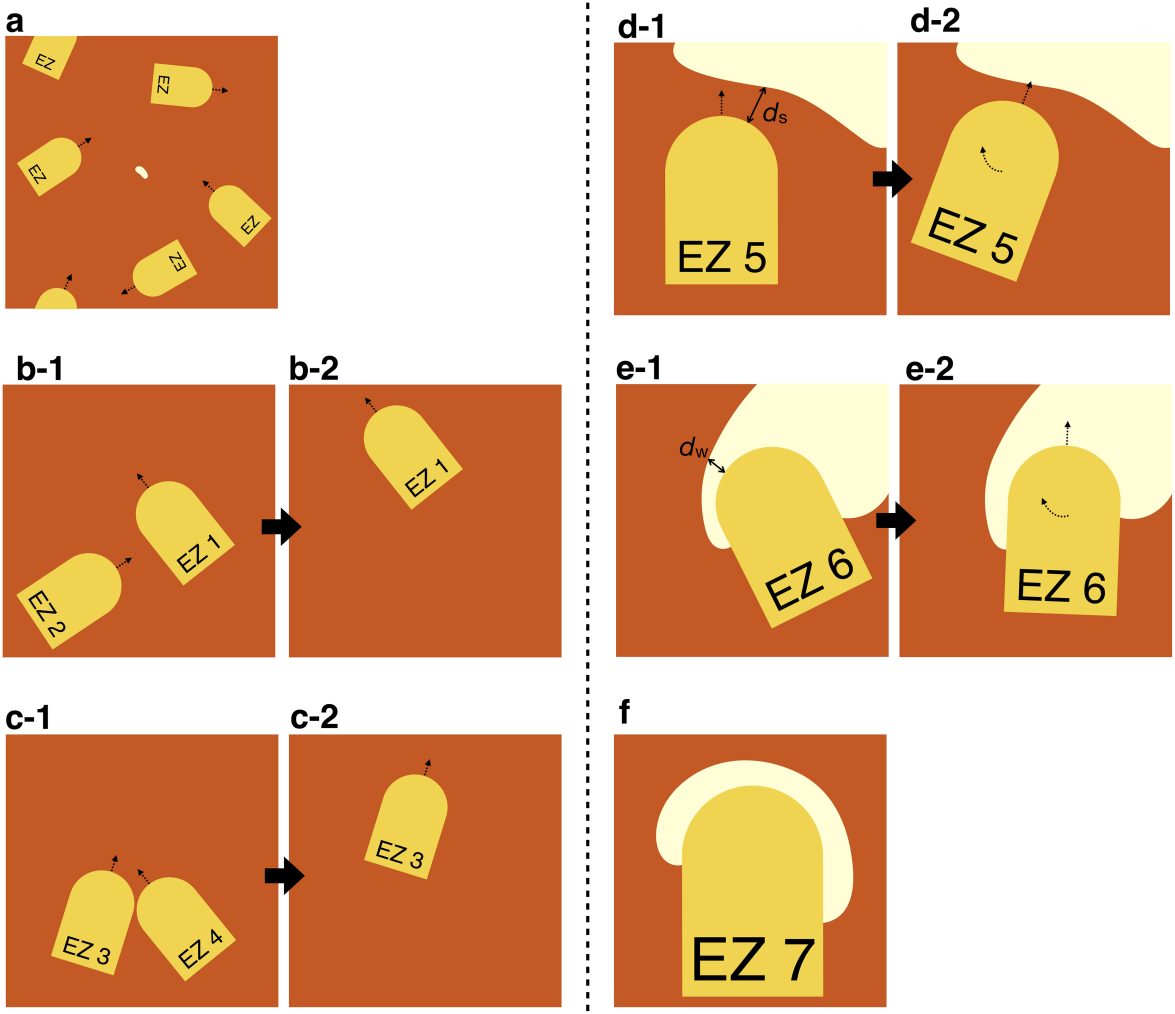
Motion of excavation zones (EZs). Schematic overview describing the EZ motion (yellow), where white regions show clusters of wax. (a) EZs can move freely in the system far from the wax. All move at the same speed. (b) When one EZ collides headfirst into another EZ, the latter (EZ 2 in this example) is instantly transported to the edge of the system. (c) When two EZs meet head on, one of the EZs (EZ 4 in this example) is instantly transported to the edge of the system. (d) When an EZ (EZ 5) detects a cluster of wax within *d*_s_, it approaches the wax by rotating its body. (e) When an EZ (EZ 6) detects wax thinner than *d*_w_, it avoids digging wax at that location by rotating its body. (f) When an EZ (EZ 7) is surrounded by thin wax, it stops in place (local equilibrium state).

Basically, in our system, EZs move ahead in a linear fashion (Fig 3a). This rule corresponds to the body section movements of the excavator. All EZs move at the same speed *v*_ex_. To approximate a bulk system by observing a small section, we assume that the boundary condition is periodic. In other words, the EZ going out of the system comes back in from the opposite side. Thus, the number of EZs is maintained by the system.

Linear uniform motion of the EZ only stops in the following three cases: (i) when one EZ touches another (Fig 3b and 3c), (ii) when an excavator finds attached wax in the vicinity before beginning excavation (EZ 5 in Fig 3d), and (iii) when it recognizes that the cluster in front is at the thinness limit (EZ 6 and EZ 7 in Fig 3e and 3f).

First, when an EZ encounters another such EZ, one of the two will be transported. That is, it will disappear and reappear at a randomly selected point on the edge of the system. When one EZ collides headfirst into the body part of another EZ, it is then transported elsewhere on the boundary and the motion of the other continues (EZ 2 in Fig 3b). When two EZs collide headfirst, one of the two EZs is transported and the motion of the other continues (for example, EZ 4 in Fig 3c). Although this “teleportation” process is non-physical, we do not think it affects the construction process, because movements away from the attached wax are irrelevant. This is also the reason why a unified EZ speed is assumed. The important point is that the EZs touch the wax cluster boundary in a random manner.

Second, when the EZs sense the wax wall within a distance *d*_s_, they approach the wax with a rotating motion (Fig 3d) that is related to the first of the two abovementioned antenna abilities.

Finally, when the wax width in front of the EZ is less than *d*_w_, the EZ does not move forward, and instead rotates to find a region that is thicker than *d*_w_ (Fig 3e). However, it does not rotate if the width of the side wax would become less than *d*_w_ as a result of the rotation. This characteristic, which originates from the second of the two abovementioned antenna abilities, prevents the EZ from penetrating the wax cluster. If the EZ is surrounded by a thin-wax region, it halts and remains in place (Fig 3f). Assuming that the thin regions are stable, this inactive EZ is regarded as being in a local equilibrium state.

### Simulation parameters

Several parameters were set in order to carry out numerical simulations involving the attachment-excavation model. The results presented in this paper were obtained from the parameter set summarized in Table 1.

**Table 1:** Parameters for numerical simulations, where *l* denotes the length of each side of the system.

Parameter		Value
EZ width	*w*_ex_	0.1*l*
EZ height	*h*_ex_	0.15*l*
Detect length	*d*_s_	0.02*l*
Minimum wax thickness	*d*_w_	0.02*l*
Area fraction	*σ*	0.15

The shape of the system used was a square with a side length of *l*, and the number of partitions was *N*_*x*_ = *N*_*y*_ = 100. As shown in Fig 2c, the shape of the EZ is characterized by the height *h*_ex_ and width *w*_ex_. The ratio of the honeybee body is roughly 1:2, which corresponds to *w*_ex_: *h*_ex_ + *w*_ex_/2; we thus set *h*_ex_ = 3*w*_ex_/2. Note that the small change of *h*_ex_ has little influence during the first stage of honeycomb construction because *h*_ex_ affects the depth of each cavity of honeycomb in later stages. The thickness parameter *d*_w_ was determined as *w*_ex_/5 from Fig 1. For the sake of simplicity, the sensitive length *d*_s_ was set equal to *d*_w_.

Another important parameter is the area fraction *σ*, which is the ratio of the total area of all EZs to the system size:

$$\begin{array}{c} \sigma =\frac{N_{\text{ex}}}{l^{2}}[\frac{\pi w_{\text{ex}}^{2}}{8}+w_{\text{ex}}(h_{\text{ex}}-\frac{w_{\text{ex}}}{2})]. \end{array}$$

In the case of small *σ*, the EZs do not touch the attached wax frequently, nor do they interact with each other. Therefore, we regard *σ* as a measure that can be used to characterize honeybee sociality. Here, it should be noted that the local density around the attached wax is larger than *σ* because of attraction from existing wax. The amount of wax attached at each time step can be controlled by the size and number of the EZs (i.e., *w*_ex_ and *σ*) under the constant supply of wax. We determine the value of *σ* in order to balance the rates of wax supply and excavation.

The angular velocity of every EZ was determined in relation to the size of each lattice cell using the following formula:

$$\begin{array}{c} \omega_{\text{ex}}=\frac{\sqrt{2}\Delta x}{h_{\text{ex}}-w_{\text{ex}}/2}. \end{array}$$

The coefficient $\sqrt{2}$ indicates the diagonal of the square. Similarly, the speed of every EZ was set as

$$\begin{array}{c} v_{\text{ex}}=\frac{\sqrt{2}}{5}\Delta x. \end{array}$$

The coefficient 1/5 was input to simplify adjustments to the excavation rate.

**Fig 4:**
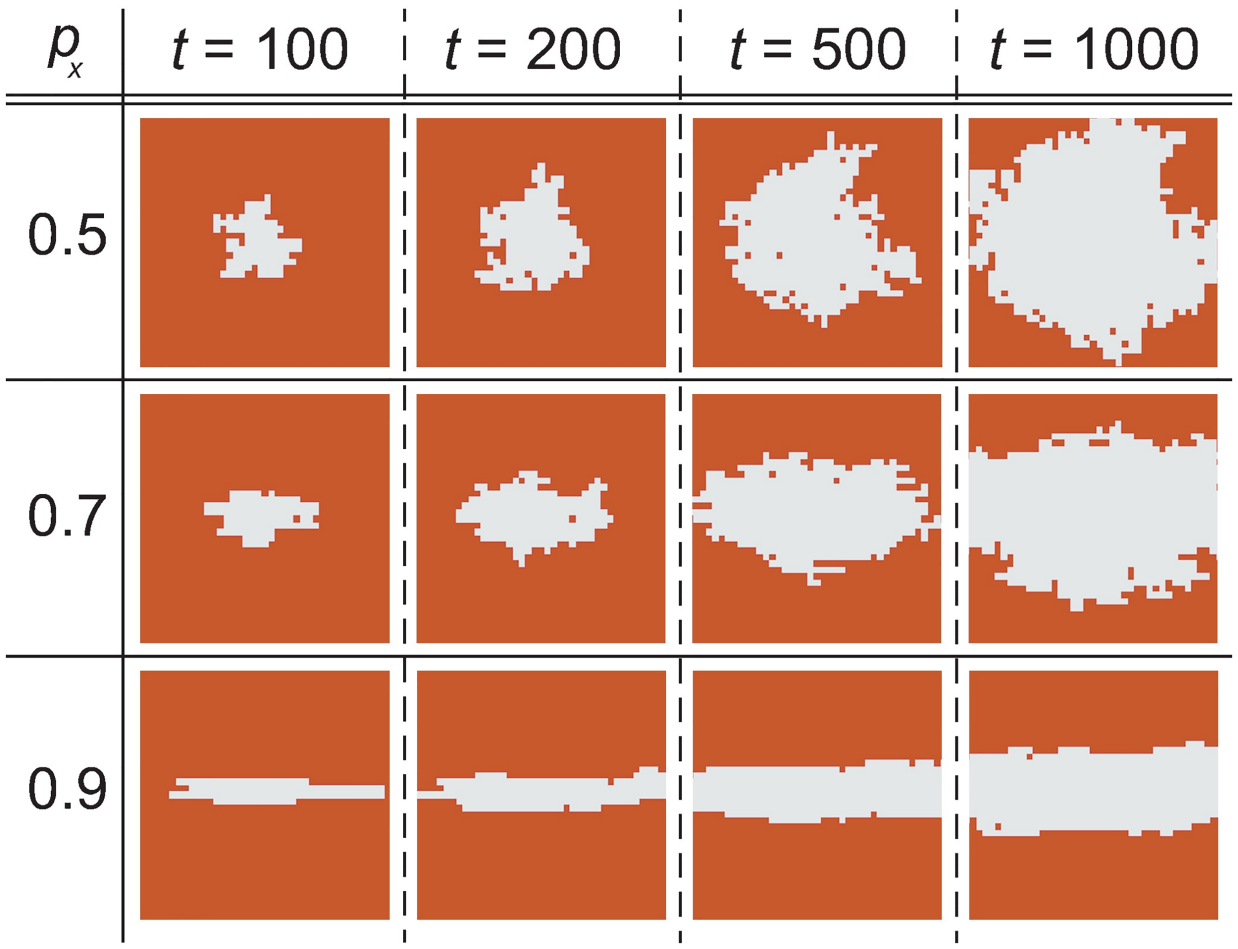
Wax growth without the excavators. Simulation results of wax growth without the excavators for different anisotropic parameters *p*_*x*_ = 0.5, 0.7, and 0.9. The system surface (i.e., off-state) is indicated by brown, and the lattice cells that contain attached wax (i.e., on-state) are shown in white. The simulation parameters are summarized in the Methods section.

As discussed in the Results section, attachers are assumed to attach wax in an anisotropic way. In other words, they recognize a certain direction (here, the *x*-direction) and they prefer to add wax in that direction. The degree of anisotropy is controlled by the probability *p*_*x*_ of growing wax in the *x*-direction [19]. The value of *p*_*x*_ = 1/2 indicates isotropic growth, and *p*_*x*_ = 1 indicates perfect anisotropic growth, which means the attached wax does not grow along the *y*-direction at all. Fig 4 demonstrates the growth of attached wax without excavators.

## Results

### Isotropic wax growth

**Fig 5:**
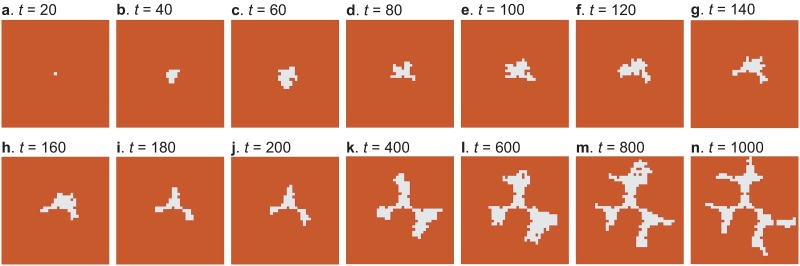
Wax growth (isotropic case). Wax growth obtained in the attachment-excavation model simulation. The frame of these results is the center of nine squares partitioned within the system. The brown area shows the surface of the system and the white region shows the attached wax. The EZs are not displayed in the figures. The simulation parameters are summarized in the Methods section. A movie showing this time evolution (with and without EZs) is uploaded as S2 Video.

Fig 5 shows the time series evolution of an attached wax cluster in which EZs are not displayed. Here, it can be seen that a tripod pattern emerges around *t* = 200. This pattern is the result of competition between wax attachment and excavation and is based on the assumption that excavators will not pierce the wax cluster. As the time advances, the wax grows from each triangle vertex and exhibits a branching pattern. This pattern can be regarded as an isotropic (2D) connection of tripods. However, it does not match that of a natural comb, which is an anisotropic (1D) linear structure as shown in Fig 1b.

Since this discrepancy suggests that an anisotropic effect is necessary, our next consideration is determining the best way to add anisotropy to our model. Noting that Belic et al. and Skarka et al. explained late stage anisotropic honeycomb construction using a mathematical model that includes interactions between worker bees [22, 23], we presume that the anisotropy originates from the polarized movement of worker honeybees near the wax cluster. Since the direction to which EZs dig depends on the local distribution of wax around them, the anisotropic movement of excavators does not significantly affect the direction. In contrast, since the attachers follow the simple rule of just adding wax to existing wax, their anisotropic movements most likely result in the anisotropic wax growth. Therefore, we keep the EZ dynamics isotropic but add anisotropy to the direction of wax growth.

### Anisotropic wax growth

**Fig 6:**
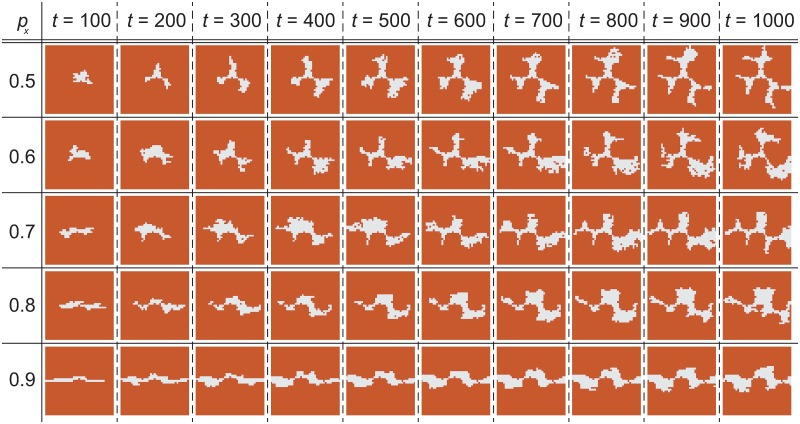
Wax growth (anisotropic cases). Wax growth obtained via the attachment-excavation model simulation for anisotropic parameters *p*_*x*_ = 0.5, 0.6, 0.7, 0.8, and 0.9, where *p*_*x*_ = 0.5 corresponds to isotropic growth. Other simulation parameters are summarized in the Methods section. The brown area denotes the system surface and the white region represents the attached wax. The EZs are not included in these figures. A movie showing this time evolution is uploaded as S3 Video.

Fig 6 shows the time evolution of an attached wax cluster for several anisotropic parameters *p*_*x*_, which is the probability of growing wax in the *x*-direction. Although the result of *p*_*x*_ = 0.6 is similar to that of *p*_*x*_ = 0.5, the results of *p*_*x*_ = 0.7 and 0.8 show the 1D connection of tripods at around *t* = 1000. Because the tendencies of these patterns are qualitatively identical if there are small changes to the control parameters such as *w*_ex_ and *σ*, we think that the pattern does not sensitively depend on the values of the control parameter. To investigate the sensitivity in detail, one should first establish a quantitative manner to evaluate the wax pattern.

**Fig 7:**
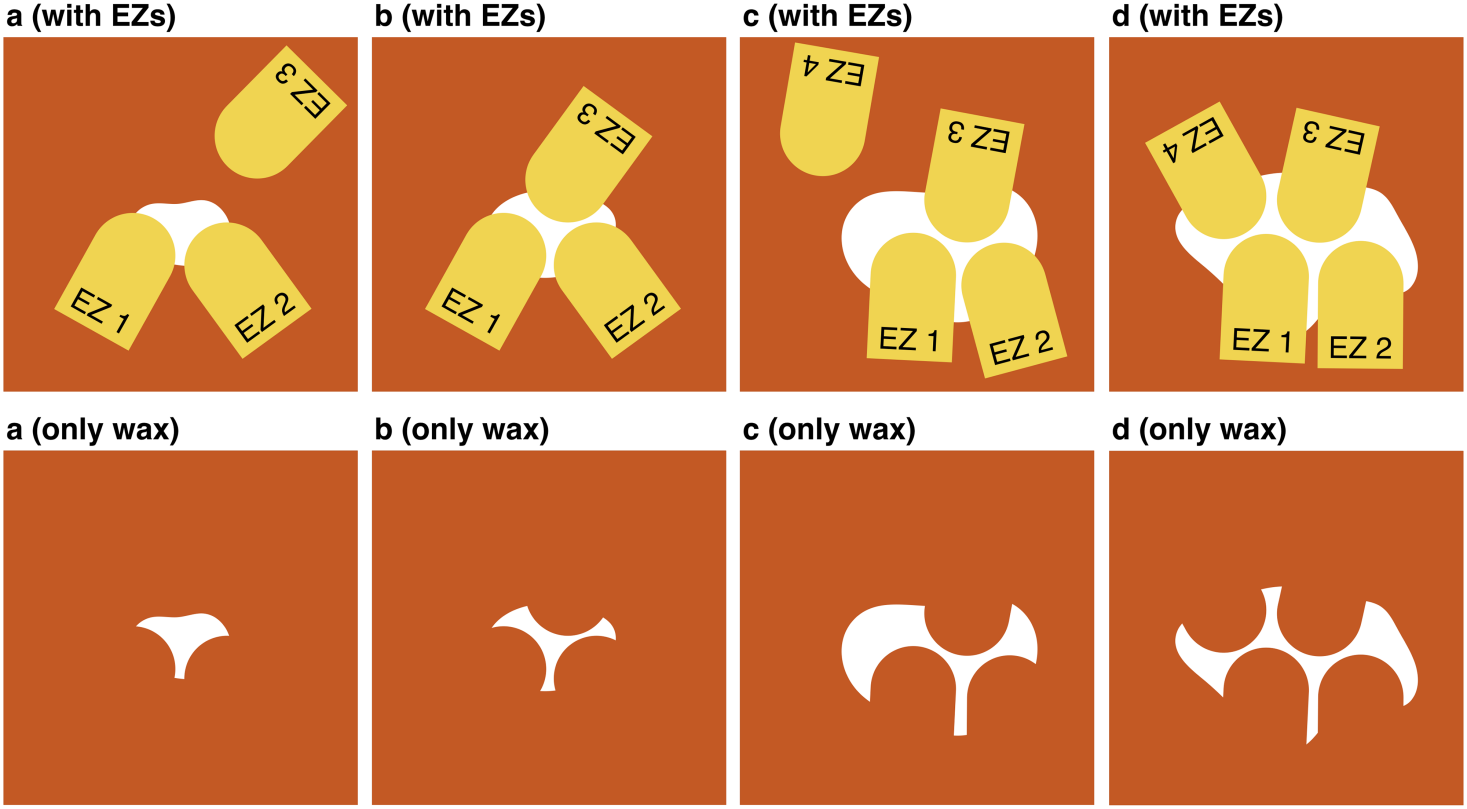
Linear sequence of constructed tripods in anisotropic cases. Schematic illustration of the tripod pattern (a-d) constructing process and their 1D connection. EZs are shown in the upper four panels but omitted from the bottom ones. The brown area denotes the surface of the system, the white region denotes the attached wax, and the yellow object denotes the EZ.

Using Fig 7 as an example, we show how anisotropy contributes to the 1D connection of the tripod patterns in the attachment-excavation model. The construction process begins when three EZs meet in the vicinity of the attached wax. The three EZs do not always arrive simultaneously, but that is irrelevant because EZs in local equilibrium states never interpenetrate. In the case of Fig 7a, EZs 1 and 2 arrive at the wax first and then begin digging. Both soon become inactive, that is, they enter a local equilibrium state because there is no thick region around them. Then, EZ 3 arrives and digs until reaching the local equilibrium state, as shown in Fig 7b.

At this point, the wax structure begins to exhibit a tripod pattern. After that, the wax continues to grow from the vertices of the tripod. Due to anisotropy, wax grows toward the left-hand side of EZ 1 and toward the right-hand side of EZ 2 in the case of Fig 7. Each EZ then rotates in order to begin excavating in a direction perpendicular to the wax growth. Next, EZ 4 arrives at the further growth part to begin work there in accordance with the movement rule illustrated by Fig 3d. As a result, the wax structure forms the 1D connection of the tripod patterns (Fig 7d), as obtained from the simulation of *p*_*x*_ = 0.7 and 0.8 around *t* = 1000. In contrast, when the wax grows isotropically, the EZs face in various directions. Therefore, the 2D-connected pattern emerges as the simulation result of *p*_*x*_ = 0.5 and 0.6.

**Fig 8:**
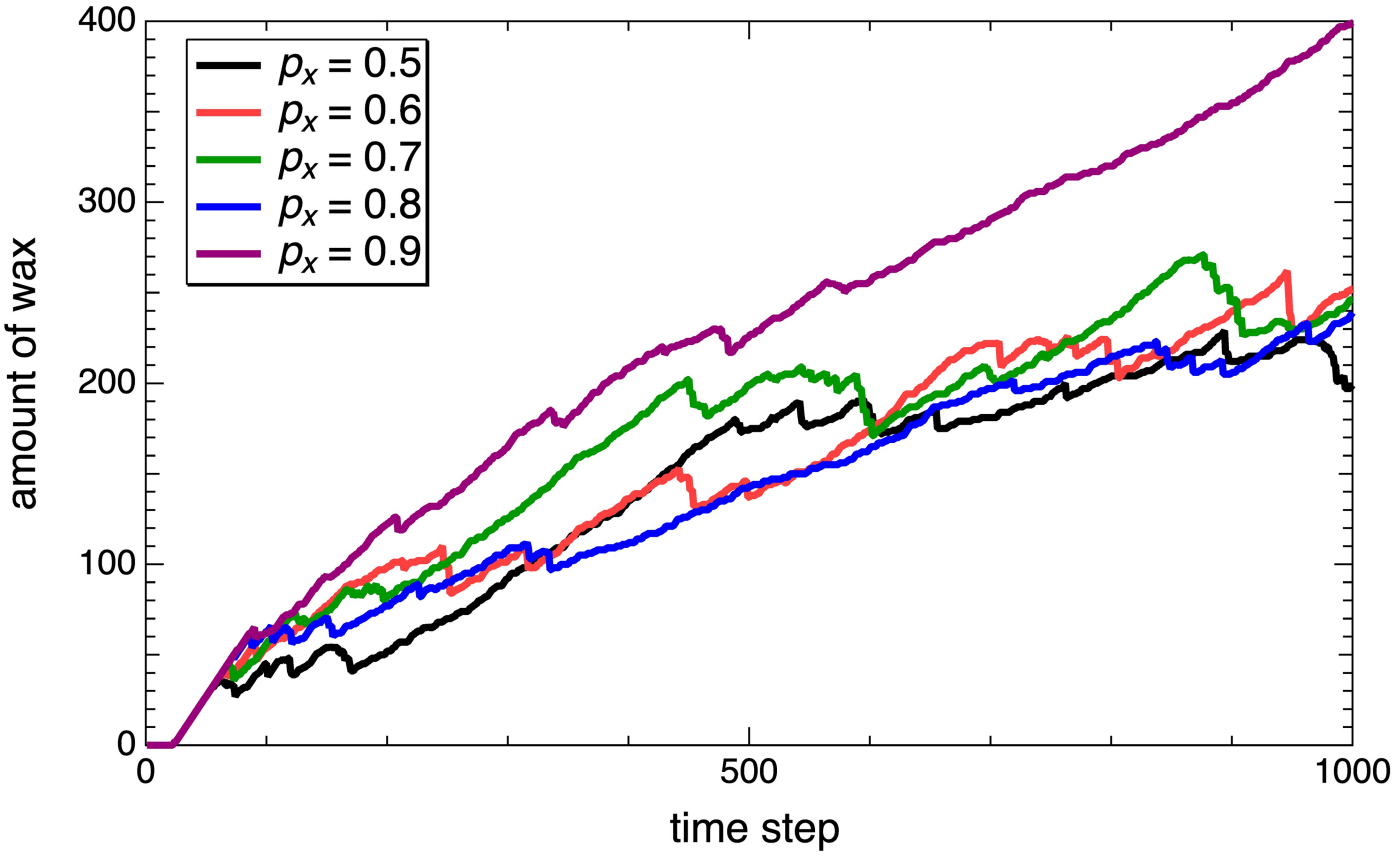
Amount of wax. Time dependence of the wax amounts for different probabilities *p*_*x*_ = 0.5, 0.6, 0.7, 0.8, and 0.9. The amounts are measured as the number of on-state lattice cells. These results correspond to Fig 6.

Fig 8 shows the amount of attached wax for different anisotropy levels. Here, we can see that, with the exception of *p*_*x*_ = 0.9, the growth rates show a similar declining behavior. This is because the EZs that exist around the wax cluster increase as the time steps increase. Note that the amount of wax increases linearly when no EZs exist. The result of *p*_*x*_ = 0.9 indicates behavior differences from those of the other *p*_*x*_. In fact, the results for *p*_*x*_ = 0.9 show a two-step construction; a slim structure at first and then the outlines of rough patterns. However, such slim structure does not appear in nature. These results suggest that the growth rate of wax in *p*_*x*_ = 0.9 is too fast to permit the construction of natural structures, and moderate wax growth anisotropy is necessary for producing the elongated structure found in actual honeycombs.

As mentioned in the Methods section, the model presumes that EZs in wax move ballistically until they stop, which differs from the behavior of actual bees. We speculate that these differences do not affect the pattern after local equilibrium, but might affect a transient pattern. It is believed that results in Fig 8 will help us to evaluate our model during comparisons with actual data.

## Discussion

Taken as a whole, our results highlight the possibility that the first process of honeycomb construction arises from self-organization. The major roles of worker bees in our model are wax attachment and excavation, which cause the inflow and outflow of wax. In other words, the system is open and non-equilibrium with regard to wax, and the patterns in this model are thus dissipative structures. Self-organization is classified into two processes: self-assembly in equilibrium states and dissipative structure formation in non-equilibrium states. Mutual assistance encourages the pattern to achieve hierarchical complexity [24]. In the first stage of honeycomb construction, the tetrapod structure is a dissipative structure and the directionally aligned structure of tetrapods can be regarded as formed through self-assembly.

Honeybees have long been considered capable of self-organization. For example, a swarm of honeybees controls its temperature as needed to reflect changes in the environment [25–27], and such thermoregulation in the honeybee swarm has been modeled by self-organization [28, 29]. In addition, honeybee colonies search for nectar sources within their foraging range and choose better ones [30], and a modeling approach has shown that the efficient concentration of effort can be interpreted as a self-organized mechanism [31]. Furthermore, comb pattern usage itself can be described by a bottom-up process [32–36]. As in the honeycomb construction process, it has been noted that honeybees benefit from self-organization [11, 22, 23, 37–40]. Our study, which is based on the first stage of honeycomb construction, presents evidence for that statement.

Further investigation is needed to clarify the role of the sense of touch, which is an important assumption in the attachment-excavation model. With regard to the imperfect honeycomb construction by honeybees with missing antennae, while we have interpreted this phenomenon as being due to the inability to perform thickness gauging, Tautz has inferred the reason as being related to the inability of antenna-damaged honeybees to measure ambient temperatures [11]. Hence, in order to validate our model, further studies will be necessary to determine how honeybees determine wax thickness via their antennae.

In our simulation, thicker cavity partitions appear in the pattern obtained for large anisotropic cases, although they have been rarely observed in nature. The anisotropy enlarges the effective supply rate in the anisotropic direction. As a result, wax growth thickens before the EZs gather around it. In addition, a higher *p*_*x*_ causes the outer perimeter of the attached wax cluster to be long. This makes it easier for EZs to touch the cluster, leading to sparse placement of EZs that dig into the wax. In contrast to our model, in which the supply rate is constant, natural honeybees most likely have a way to regulate the supply rate in order to avoid creating such lumps.

Our model could also be improved by adding a function that allows the reuse of wax excised by EZs. This is important because, although wax attachment is independent of the excavation process in our current model, an abovementioned experiment using colored wax revealed that the wax honeybees excise from one location can be reattached in other locations [20].

From a model improvement viewpoint, interactions between EZs may also need reconsideration. However, it is unclear how any changes would affect pattern formation. Since workers do not work concurrently to build a honeycomb cell, some honeybees will excise wax independently of the other bees. Therefore, it will be necessary to include bee-bee interactions in the wax growth process rather than focus solely on EZ movements.

## Conclusion

In summary, in an attempt to gain a better understanding of the first stage of honeycomb construction, we proposed an agent-based model as the attachment-excavation model, in which the roles of worker honeybees are modeled into the growth of beeswax and the dynamics of EZs. Since the workers act according to simple rules, our model does not require them to have any prior knowledge of the complex shape that they build. Using the numerical simulation for this model, the tripod structure, which is the basic building block of the honeycomb structure in a 2D reduction, was the result of competition between the wax-attaching and wax-removing workers. Thus, the tripod structure can be regarded as a dissipative structure. In addition, by supplying wax unidirectionally, the anisotropic (1D) connection of tripod patterns has been also obtained. We can conclude that the first stage of honeycomb construction can be understood in terms of self-organization, the formation of tetrapod structures (dissipative structure), and their 1D connections (self-assembly). Taken together, these give rise to the hierarchical complexity of honeycomb. We anticipate that our study will pave the way towards understanding the achievement of complexity within a hierarchy. Moreover, since it is widely known that honeycomb structures are strong, can be constructed at reduced material costs, and have high storage capacities, the simple algorithm we have proposed herein has the potential to contribute to the bottom-up construction of other honeycomb structure types.

## Supporting information

S1 Video Observations of the first honeycomb construction process. (MP4)

S2 Video Isotropic wax growth case. Animation corresponding to Fig 5.
(MP4)

S3 Video Anisotropic wax growth cases. Animation corresponding to Fig 6.
(MP4)
